# Mapping the knowledge and understanding of menarche, menstrual hygiene and menstrual health among adolescent girls in low- and middle-income countries

**DOI:** 10.1186/s12978-017-0293-6

**Published:** 2017-03-01

**Authors:** Venkatraman Chandra-Mouli, Sheila Vipul Patel

**Affiliations:** 1 Department of Reproductive Health and Research, World Health Organization, Avenue Appia 20, 1211 Geneva 27, Switzerland; 2 Department of Health Policy and Management, Gillings School of Global Public Health, University of North Carolina at Chapel Hill, 135 Dauer Drive, Chapel Hill, NC 27599 USA

**Keywords:** Menarche, Menstruation, Menstrual health, Menstrual health problems, Menstrual hygiene management, Adolescent girls

## Abstract

**Background:**

Menstruation is a natural physiological process that requires proper management. Unlike other normal bodily processes, menstruation is linked with religious and cultural meanings that can affect the perceptions of young girls as well as the ways in which the adults in the communities around them respond to their needs.

**Objectives:**

This review aims to answer the following questions: (1) how knowledgeable are adolescent girls in low- and middle-income countries about menstruation and how prepared are they for reaching menarche, (2) who are their sources of information regarding menstruation, (3) how well do the adults around them respond to their information needs, (4) what negative health and social effects do adolescents experience as a result of menstruation, and (5) how do adolescents respond when they experience these negative effects and what practices do they develop as a result?

**Methods:**

Using a structured search strategy, articles that investigate young girls’ preparedness for menarche, knowledge of menstruation and practices surrounding menstrual hygiene in LMIC were identified. A total of 81 studies published in peer-reviewed journals between the years 2000 and 2015 that describe the experiences of adolescent girls from 25 different countries were included.

**Results:**

Adolescent girls in LMIC are often uninformed and unprepared for menarche. Information is primarily obtained from mothers and other female family members who are not necessarily well equipped to fill gaps in girls’ knowledge. Exclusion and shame lead to misconceptions and unhygienic practices during menstruation. Rather than seek medical consultation, girls tend to miss school, self-medicate and refrain from social interaction. Also problematic is that relatives and teachers are often not prepared to respond to the needs of girls.

**Conclusion:**

LMIC must recognize that lack of preparation, knowledge and poor practices surrounding menstruation are key impediments not only to girls’ education, but also to self-confidence and personal development. In addition to investment in private latrines with clean water for girls in both schools and communities, countries must consider how to improve the provision of knowledge and understanding and how to better respond to the needs of adolescent girls.

## Plain English summary

Our paper maps the knowledge, attitudes, beliefs and practices surrounding menarche, menstrual hygiene and menstrual health among adolescent girls in low and middle income countries in order to inform the future design of relevant policies and programming.

Our study of over 80 journal articles from a number of low and middle income countries confirmed that:

- Many adolescent girls start their periods uninformed and unprepared
- Mothers are the primary source of information, but they inform girls too-little and too-late and often communicate their own misconceptions
- Because menstruation is widely seen as polluting and shameful, girls are often excluded and shamed in their homes and in their communities
- Many do not have the means for self-care and do not get the support they need when they face problems, which hinders their ability to carry on with everyday activities and may also establish a foundation for life-long disempowerment

Efforts to respond to girls’ needs are fragmented and piece-meal. There is growing acknowledgement that efforts are more likely to be successful if they come together in a whole-of-community approach that involves schools, health facilities, and homes and communities to:

- Educate girls about menstruation
- Create norms that see menstruation as healthy and positive, not shameful and dirty
- Improve access to sanitary products, running water, functional toilets and privacy for self-care
- Improve care for and support by girls’ families when they have their periods
- Improve access to competent and caring health workers when they experience menstrual health problems

## Background

Girls in many low- and middle-income countries (LMIC) enter puberty with knowledge gaps and misconceptions about menstruation, unprepared to cope with it and unsure of when and where to seek help. This is because the adults around them, including parents and teachers, are themselves ill-informed and uncomfortable discussing sexuality, reproduction and menstruation (which frequently comes laden with dirty, polluting and shameful connotations).

To respond to the increased international attention on empowering girls through the United Nation’s Sustainable Development Goals, this review aims to map the knowledge, attitudes, beliefs and practices surrounding menarche, menstrual hygiene and menstrual health among adolescent girls in LMIC in order to inform the future design of relevant policies and programming. To do this, our objectives are to answer the following questions: (1) how knowledgeable are girls in LMIC about menstruation and how prepared are they for reaching menarche, (2) who are their sources of information regarding menstruation, (3) how well do the adults around them respond to their information needs, (4) what negative health and social effects do adolescents experience as a result of menstruation, and (5) how do adolescents respond when they experience these negative effects and what practices do they develop as a result?

## Methods

Our literature search aimed to identify articles that evaluated the knowledge of girls regarding menstruation, their information sources, the health and social effects of menstruation, and how adolescents and adults responded to these effects. We searched Google Scholar, PubMed and EBSCO’s Global Health database for articles in peer-reviewed journals published between 2000 and 2015. To identify relevant literature, we used the following search strategy: (menarche or menstruation or menstrual health or menstrual hygiene or menstrual management) and (adolescence or adolescent or youth or young) and (female or girl or women) and (knowledge or belief or practice or experience).

Through a title and abstract review, papers in English that addressed the experiences of adolescent girls (ages 10–19) in LMIC were retained. Full text articles were reviewed to determine whether studies addressed one or more of our five questions. Given the limited research available, descriptive overviews and interventions using quantitative, qualitative, or mixed methods of any sample size were all included.^1^ While the focus of this paper is on menstrual experiences, studies that reported on the preparedness and attitudes of pre-menarcheal girls were included so long as data were stratified by those who had and had not reached menarche. To complement our search, we reviewed the reference lists of the included articles and identified a small number of additional studies that met these broad criteria. Finally, we searched and included publications by United Nations agencies and international non-governmental organizations that responded to how organizations and their LMIC partners are responding to the needs of girls.

## Results

A total of 81 articles were identified after discarding duplicate articles and those that did not meet inclusion criteria (Table 1).

**Table 1:** Study characteristics

Characteristic	Frequency
Design	
Descriptive	70
Intervention	11
Method	
Mixed methods	16
Qualitative	7
Quantitative	58
Region	
East Africa (Ethiopia, Kenya, Malawi, Tanzania, Uganda)	10
North Africa (Egypt)	4
West Africa (Ghana, Nigeria)	10
North/Central America (Mexico)	3
South America (Brazil)	1
East Asia (China)	1
Southeast Asia (Malaysia)	6
South Asia (Bangladesh, India, Nepal, Pakistan, Sri Lanka)	39
West Asia (Iran, Jordan, Lebanon, Turkey)	7
Setting^a^	
Mix	21
Rural	23
Urban	30
School status^a^	
Mix of school-going and out-of-school	12
School-going	63

^a^All included studies did not specify setting or girls’ school status

### How knowledgeable are girls about menstruation and how prepared are they for reaching menarche?

Girls across LMIC have limited knowledge and understanding about menstruation prior to reaching menarche. The proportion of girls that were aware ranged from 2.8% of rural girls questioned in Rajasthan, India [1] to all urban girls in Turkey [2] (Table 2). Village-based meetings for girls in a Maharashtra, India were tested as a platform for disseminating health messages, and significantly contributed to an increase from 35.1% of girls interviewed in 2003 to 55.4% of girls interviewed in 2007 being aware of menstruation before its onset (*p*-value < 0.05) [3].

**Table 2:** Awareness of menstruation prior to menarche

First author, Year	Country	Setting	School status	N	Aware
North Africa					
Eswi 2012 [45]	Egypt	Urban	School-going	200	74.0%
South Asia					
Bosch 2008 [28]	Bangladesh	Rural	Unclear	156	35.0%
Khanna 2005 [1]	India	Mix	Out-of-school	358	5.6%
Dambhare 2012 [32]	India	Mix	School-going	561	75.6%
Juyal 2013 [65]	India	Mix	School-going	453	64.5%
Khanna 2005 [1]	India	Mix	School-going	372	9.8%
Thakre 2011 [12]	India	Mix	School-going	387	37.0%
Khanna 2005 [1]	India	Rural	Mix	NR^a^	2.8%
Dasgupta 2008 [8]	India	Rural	School-going	160	67.5%
Shanbhag 2012 [10]	India	Rural	School-going	329	57.9%
Sudeshna 2012 [15]	India	Rural	School-going	190	47.4%
Dhingra 2009 [29]	India	Rural	Unclear	200	64.0%
Tiwari 2006 [11]	India	Unclear	School-going	763	62.7%
Khanna 2005 [1]	India	Urban	Mix	NR^a^	12.1%
Omidvar 2010 [46]	India	Urban	School-going	336	64.5%
Yasmin 2013 [13]	India	Urban	School-going	147	42.2%
Bobhate 2011 [7]	India	Urban	Unclear	241	20.3%
Udgiri 2010 [23]	India	Urban	Unclear	342	18.4%
Ali 2010 [6]	Pakistan	Urban	Government school	425	47.8%
Ali 2010 [6]	Pakistan	Urban	Out-of-school	425	38.8%
Ali 2010 [6]	Pakistan	Urban	Private school	425	34.1%
West Asia					
Reis 2011 [27]	Turkey	Urban	Mix	310	67.4%
Ersoy 2004 [2]	Turkey	Urban	School-going	1017	100.0%

*NR* Not reported

^a^Of 730 girls, the number in rural versus urban settings was not specified

Three quarters of 1,573 Chinese girls surveyed rated their menstrual knowledge as inadequate or very inadequate [4]. Even so, girls with any knowledge often hold misconceptions about menstruation. For example, a study conducted in rural Nepal reported that 6.0% of 150 girls surveyed recognized menstruation as a physiological process while 82.0% believed it was a curse [5]. Understanding that menstruation is a natural bodily function was higher at 19.3% in Pakistan [6], 18.3–86.3% in five Indian states [1, 7–13], and 96.7% in Nigeria [14]. Menstruation was considered a curse, disease, or representation of sin by some girls in five Indian states [7–12, 15] and Uganda [16]. Prior to receiving health education at school, 72.4% girls in India considered menstrual blood impure [17].

An additional knowledge gap among girls is a lack of awareness regarding the origins of menstrual blood—no more than a third of girls correctly identified the uterus as the source of menstrual blood in four Indian states [7, 12, 15, 18, 19] and rural Nepal [5]. One study in a fourth Indian state reported almost no girls being aware of the source of their blood (2.5%) [8], while another nearly two-thirds being aware (63.3%) [13]. In cities in Pakistan [6] and Nigeria [14], 37.2 and 78.7% identified the uterus as the source, respectively, compared to 82.9% of school-going girls in rural Uganda [16]. Only a third of rural-living, high school girls surveyed in India associated the attainment of menarche with the capacity to conceive [10].

Age had a significant influence on slum dwellers’ knowledge in India, with older girls more knowledgeable about menstruation than their younger counterparts (*p*-value <0.05) [7]. Similar findings were reported among Nigerian schoolgirls (*p*-value <0.05) [20]. Compared to those not attending school, awareness was greater among schoolgirls in India [1] and Pakistan [6]. Education level had a significant influence on menstrual knowledge in India [7] and Nigeria [14] (*p*-value <0.05).

### Who are girls’ sources of information?

Across LMIC studied, mothers were often the most frequently cited source of information and advice for girls regarding menstruation (Table 3). Compared to girls residing in urban parts of Ethiopia [21] and India [22], those in rural settings reported their mothers as an information source less often (possibly because there were other female relatives they could turn to). Following mothers, sisters were the next most common resource in four Indian states [1, 11, 12, 23], Mexico [24], Nepal [25], Nigeria [20, 26], Pakistan [6], and Turkey [27], though they were utilized by less than a quarter of girls. In some contexts, sisters and friends surpassed mothers as the primary source of information [6, 13, 28, 29].

**Table 3:** Most commonly reported sources of menstrual information

First author, Year	Country	Setting	School status	N	Most common source (%)
East Africa					
Zegeye 2009 [21]	Ethiopia	Mix	School-going	564	Mother (39.7%)
North Africa					
Abd El-Hameed 2011 [44]	Egypt	Mix	School-going	160	Mother (59.4%)
El-Gilany 2005 [30]	Egypt	Mix	School-going	642	Mother (92.2%), mass media (92.2%)
Eswi 2012 [45]	Egypt	Urban	School-going	200	Mother (53.0%)
West Africa					
Gumanga 2012 [31]	Ghana	Urban	School-going	456	Parent (80.2%)
Adinma 2009 [26]	Nigeria	Urban	School-going	550	Mother (48.4%)
Ajah 2015 [42]	Nigeria	Urban	School-going	482	Mother (81.5%
Aniebue 2009 [40]	Nigeria	Urban	School-going	495	Mother (71.5%)
Lawan 2010 [20]^f^	Nigeria	Urban	School-going	385	Mother (35.3%)
Oche 2012 [14]	Nigeria	Urban	School-going	122	Mother or grandmother (56.6%)
Central America					
Marván 2012 [24]^f^	Mexico	Urban	School-going	405	Mother (78.0%)
South Asia					
Bosch 2008 [28]	Bangladesh	Rural	Unclear	86	Sister (29.0%)
Dambhare 2012 [32]^a^	India	Mix	School-going	561	Mother (38.2%)
Thakre 2011 [12]^a^	India	Mix	School-going	143	Mother (71.3%)
Khanna 2005 [1]	India	Rural	Mix	-	Mother (55.1%)
Dasgupta 2008 [8]^a^	India	Rural	School-going	160	Mother (37.5%)
Kanotra 2013 [92]	India	Rural	School-going	323	Mother (94.4%)
Kotecha 2009 [33]^a^	India	Rural	School-going	340	Mother (32.9%)
Mudey 2010 [34]	India	Rural	School-going	300	Mother (40.7%)
Shanbhag 2012 [10]	India	Rural	School-going	506	Mother (55.1%)
Sudeshna 2012 [15]^a^	India	Rural	School-going	80	Mother or sister (45.0%)
Dhingra 2009 [29]	India	Rural	Unclear	200	Friend (83.0%)
Tiwari 2006 [11]	India	Unclear	School-going	486	Mother (60.7%)
Khanna 2005 [1]	India	Urban	Mix	-	Mother (66.8%)
Sharma 2008 [64]^b^	India	Urban	Mix	156	Mother (73.7%)
Omidvar 2010 [46]^a^	India	Urban	School-going	215	Mother (54.0%)
Yasmin 2013 [13]	India	Urban	School-going	147	Friend (20.4%)
Bobhate 2011 [7]^c^	India	Urban	Unclear	241	Mother (75.9%)
Udgiri 2010 [23]^a^	India	Urban	Unclear	63	Mother (63.5%)
Adhikari 2007 [5]	Nepal	Rural	School-going	150	Coursebook (14.7%)
Sharma 2003 [25]	Nepal	Urban	School-going	96	Mother (37.5%)
Ali 2010 [6]^a^	Pakistan	Urban	Government school	203	Sister (35.5%)
Ali 2010 [6]^a^	Pakistan	Urban	Out-of-school	165	Sister (49.7%)
Ali 2010 [6]^a^	Pakistan	Urban	Private school	145	Mother (37.9%)
Chandraratne 2011 [43]^b^	Sri Lanka	Urban	School-going	473	Mother (67.0%)
Southeast Asia					
Lee 2006 [37]	Malaysia	Mix	School-going	2247	Mother (80.0%)
Wong 2011 [47]^e^	Malaysia	Rural	School-going	984	Mother (62.3%)
Wong 2011 [38]^d^	Malaysia	Rural	School-going	577	Mother (31.7%)
Wong 2011 [48]^d^	Malaysia	Urban	School-going	407	Mother (62.7%)
West Asia					
Jarrah 2012 [36]	Jordan	Urban	School-going	408	Mother (57.1%)
Reis 2011 [27]	Turkey	Urban	Mix	310	Mother (18.7%)
Ersoy 2004 [2]	Turkey	Urban	School-going	1017	Mother (55.7%)

^a^Pre-menarcheal sources

^b^Sources among those with menstrual problems

^c^Post-menarcheal sources

^d^Sources among those who ever received information

^e^Sources among those with dysmenorrhea

^f^First source

A majority of studies which examined the roles of teachers and/or health professionals as providers of menstrual information ranked them as the least common sources compared to female relatives and friends (Egypt [30], Ghana [31], India [1, 7, 11, 12, 15, 32–35], Jordan [36], Malaysia [37–39], Nepal [25], Nigeria [14, 20, 26, 40–42], Sri Lanka [43], and Turkey [27].) Teachers were cited as a source by less than 5.0% of girls questioned in three Indian states [1, 32, 33], Nepal [25], and Sri Lanka [43]. At most, a third of subjects in urban Nigeria cited teachers as a source [41]. While students in urban Malaysia were more likely than those in rural settings to cite teachers as a source, a considerable number had never encountered menstrual-related topics in school [39]. Less than 1.0% of girls in a rural part of India [33] and urban parts of Jordan [36] reported having received information regarding menstruation from health professionals. At most, a quarter of study participants in urban Nigeria cited health professionals as a source [41].

Some studies reported mass media, such as radio, television, newspapers, magazines, books, and the Internet either as the only resource available to girls or as supplements to other sources of information (Egypt [30, 44, 45], Ghana [31], India [15, 29, 32, 34, 35, 46], Jordan [36], Malaysia [37–39, 47, 48], Nepal [25], Nigeria [40–42], Sri Lanka [43], and Turkey [2, 27]). In a few instances, such sources were reported by more than a quarter of girls: 72.4% in Nigeria [41], 92.2% in Egypt [30], and 29.2–43.6% in Malaysia [37, 38, 47, 48].

Girls reported not having received information from any source in some studies. As few as 6.8 and 7.0% of girls in urban Nigeria [42] and Egypt [45], respectively, and 7.8% of a mix of urban- and rural-living girls in Ethiopia [21] reported having no source. A study of urban- and rural-living girls in India reported a quarter without a source [32]. In rural Nepal, 76.0% of girls reported having no menstrual information source [5].

### How well do adults respond to girls’ information needs?

Whether by a relative, friend, or other community member, the information on menstrual health and hygiene provided to adolescents is not always timely nor is it adequate.

Researchers found that mothers and other relatives in India [1, 7] and Tanzania [49] who did provide girls with information often did not do so until after menarche. In Mexico, however, 94.0% of girls reported that they had discussed menstruation with their mothers prior to menarche [24]. A study in Nigeria reported 55.2% of school-going girls were “trained” prior to reaching menarche, which included being made aware of what to expect at menarche and how to collect blood and dispose of materials [40]. Further, parents’ education level was found to have a significant influence on pre-menarcheal knowledge in Nigeria (*p*-value <0.05) as girls whose parents had received tertiary education were the most likely to have been trained [40]. In India [15, 33] and Kenya [50, 51], girls reported that little information about menstruation was provided and nearly no explanation. Sources of information may have their own misconceptions about menstruation, which they may pass on. Mothers interviewed in Bangladesh attributed menstruation to God [28]. During initiation rites in Malawi, misconceptions, like men can get hurt if they come in contact with menstrual blood, are told to girls by female relatives [52].

Given the link between menstruation and the ability to conceive, mothers interviewed in Bangladesh did not consider it appropriate to discuss the matter with their pre-menarcheal daughters [28]. Both mothers and teachers, most of whom were male, in Kenya cited discomfort as an impediment to discussing menstruation with girls [53]. Teachers in rural Tanzania warned their students that their mothers would be very upset if told about their reaching menarche [49]. This may be a result of cultural taboos that prevent parents from discussing sex-related topics with their daughters. Taboos were also cited as a by the few teachers in Tanzania who wanted to provide support to their students [54]. Teachers in Kenya did not perceive menstrual education as part of their role nor did they feel properly prepared to share information with their students [53].

A majority of teachers (70–90%) at schools in Ghana who had been trained to use play-based approaches to promote menstrual knowledge and practices were confident in discussing menstruation with their students compared to their counterparts at schools not using similar approaches who had limited conversations [55]. Overall, 82.4% of study participants in Jordan felt they were not adequately prepared for reaching menarche [36]. Of girls in rural Nepal who received information from a parent, friend or a coursebook, an overwhelming majority felt menstrual-related topics were not properly taught [5]. Four-fifths of school-going girls questioned in Egypt wanted more information [30]. However, girls in Malaysia [39] and Tanzania [54] reported feeling ashamed, embarrassed and uncomfortable when inquiring about menstruation from adults.

### What are the emotional, physical and social impacts of menstruation on girls?

Anticipated and experienced menstruation-related impacts were mostly negative (with some positive impacts when girls were better informed/prepared). While eight out of every ten study participants in Mexico expected at least one positive change to occur upon reaching menarche, all expected at least one negative change to occur; the nine most expected changes among urban and rural girls were negative, e.g., discomfort, worry, cramps [56]. Overall, 89.4% of anticipated changes reported by pre-menarcheal girls and 88.7% of experienced changes reported by post-menarcheal girls were negative [57].

### Emotional impacts

A quarter to eight out of every ten girls questioned in various LMIC reported not being mentally prepared for reaching menarche (Brazil [58], China [4], India [11], Jordan [36], Mexico [24, 56], Nepal [25], Nigeria [40],). Many girls had negative reactions to their first period (Table 4). For example, a majority of school-going girls in one study in India described menarche as a shocking or fearful event and many cried upon seeing their blood [18].

**Table 4:** Negative reaction upon reaching menarche

First author, Year	Country	Setting	School status	N	Negative reaction^a^ (%)
West Africa					
Aniebue 2009 [40]	Nigeria	Urban	School-going	495	50.3%
Oche 2012 [14]	Nigeria	Urban	School-going	122	53.3%
Central America					
Marván 2001 [57]	Mexico	Urban	School-going	98	15.3%
East Asia					
Tang 2003 [4]	China	Unclear	School-going	1,573	72.0%
South Asia					
Bosch 2008 [28]	Bangladesh	Rural	Unclear	86	64.0%
Mudey 2010 [34]	India	Rural	School-going	300	43.7%
Shanbhag 2012 [10]	India	Rural	School-going	329	44.1%
Tiwari 2006 [11]	India	Unclear	School-going	763	20.6%
Bobhate 2011 [7]	India	Urban	Unclear	241	64.7%
Udgiri 2010 [23]	India	Urban	Unclear	342	31.0%
Adhikari 2007 [5]	Nepal	Rural	School-going	150	96.7%
Ali 2010 [6]	Pakistan	Urban	Government school	425	55.8%
Ali 2010 [6]	Pakistan	Urban	Out-of-school	425	62.6%
Ali 2010 [6]	Pakistan	Urban	Private school	425	55.1%
West Asia					
Reis 2011 [27]	Turkey	Urban	Mix	310	43.9%
Ersoy 2004 [2]	Turkey	Urban	School-going	1,017	49.8%

^a^Reported shock, panic, confusion, tension, fear, shame or embarrassment at menarche

Some school-going girls perceived menstrual blood to be dirty or described feeling disgusted by their period: 30.5% in Lebanon [59], 48.9% versus 72.8% in rural versus urban Malaysia [38, 48], and 10.0–23.4% in two Indian states [11, 17, 34]. Additionally, girls in Kenya revealed that “the girl with her period is the one to hang her head” because she becomes the target of unwanted and sometimes unkind attention [53]. Mood swings and irritability connected to menstruation were both reported by more than two-thirds of schoolgirls in India [60], Lebanon [59], and Malaysia [38, 48].

Not all feelings about reaching menarche were negative; more than half of schoolgirls questioned in China [4], India [9], and Malaysia [38, 48] felt pride in maturing. Focus groups in rural and urban settings in Kenya [51] and Tanzania [61] with female students revealed a similar sentiment. The more school-going girls in Mexico knew about menstruation, the less negative their attitudes (*p*-value < 0.05); and the more prepared they felt, the more positive their attitudes (*p*-value < 0.0001) [24]. A later menarcheal age and higher socioeconomic status seemed to further reduce negative reactions among girls in Malaysia [39] and Turkey [2], respectively.

### Physical impacts

Physical impacts of menstruation that were commonly reported across studies included premenstrual symptoms or syndrome and painful periods. These outcomes were almost always reported by at least half of the sample (Table 5). At most, 93.2% of rural-living girls in India reported experiencing a premenstrual symptom [62] and 94.4% of school-going girls in Egypt reported experiencing dysmenorrhea [44]. In Ethiopia, girls with premenstrual symptoms suffered more often from dysmenorrhea than those without (82.4% versus 40.3%, respectively) [21]. Of girls with dysmenorrhea in Ghana, nearly two-thirds experienced symptoms during most or all cycles [31]. A majority of rural-living girls surveyed in Malaysia considered dysmenorrhea a normal aspect of menstruation [47].

**Table 5:** Physical impacts of menstruation

First author, Year	Country	Setting	School status	N	PMS^a^	Severe pain^b^	Headache	Swelling^c^	Fatigue^d^
East Africa									
Zegeye 2009 [21]	Ethiopia	Mix	School-going	565	75.4%	72.0%	NR	NR	NR
North Africa									
Abd El-Hameed 2011 [44]	Egypt	Mix	School-going	160	NR	94.4%	NR	NR	NR
West Africa									
Gumanga 2012 [31]	Ghana	Urban	School-going	456	NR	74.4%	NR	NR	NR
Ajah 2015 [42]	Nigeria	Urban	School-going	482	75.1%	64.1%	NR	NR	NR
Aniebue 2009 [40]	Nigeria	Urban	School-going	495		26.7%	NR	NR	NR
Central America									
Marván 2001 [57]	Mexico	Urban	School-going	98	NR	NR	6.1%	NR	14.3%
South America									
Pitangui 2013 [58]	Brazil	Urban	School-going	174	NR	73.0%	14.4%	61.5%	27.6%
South Asia									
Dambhare 2012 [32]	India	Mix	School-going	561	56.3%	56.2%	26.7%	NR	NR
Thakre 2012 [22]	India	Mix	School-going	387	55.8%	61.0%	NR	NR	NR
Baidya 2014 [77]	India	Rural	Mix	200	8.5%	59.5%	16.0%	NR	4.0%
Rana 2015 [63]	India	Rural	Mix	400	NR	46.8%	NR	NR	NR
Bodat 2013 [67]	India	Rural	School-going	622	NR	58.1%	NR	NR	NR
Kanotra 2013 [92]	India	Rural	School-going	323	3.1%	18.3%	NR	NR	NR
Mudey 2010 [34]	India	Rural	School-going	300	NR	NR	25.7%	NR	NR
Shanbhag 2012 [10]	India	Rural	School-going	329	NR	61.3%	NR	NR	NR
Wasnik 2015 [72]	India	Rural	School-going	435	17.9%	62.3%	6.7%	NR	NR
Patil 2013 [62]	India	Rural	Unclear	440	93.2%	28.0%	1.8%	NR	NR
Chaudhuri 2012 [60]^e^	India	Unclear	School-going	224	NR	59.8%	28.1% (of 128)	NR	70.3% (of 128)
Sharma 2008 [64]	India	Urban	Mix	198	63.1%	67.2%	16.7%	11.1%	48.0%
Chandraratne 2011 [43]^f^	Sri Lanka	Urban	School-going	594	66.2%	61.3%	28.1% (of 393)	NR	29.1% (of 393)
Nair 2012 [73]	India	Urban	School-going	3443	NR	72.4%	13.9%	NR	36.1%
Sharma 2003 [25]	Nepal	Urban	School-going	96	NR	69.8%	NR	NR	NR
Southeast Asia									
Lee 2006 [37]	Malaysia	Mix	School-going	2247	74.7%	69.3%	NR	NR	NR
Wong 2011 [47]	Malaysia	Rural	School-going	1295	NR	76.0%	NR	NR	NR
Wong 2011 [38]	Malaysia	Rural	School-going	1295	63.1%	NR	47.3%	12.6%	81.1%
Wong 2010 [78]	Malaysia	Urban	School-going	1075	NR	74.5%	NR	NR	NR
Wong 2011 [48]	Malaysia	Urban	School-going	1076	56.5%	NR	38.4%	13.1%	75.4%
West Asia									
Poureslami 2002 [79]	Iran	Urban	School-going	250	NR	71.2%	NR	NR	NR
Jarrah 2012 [36]	Jordan	Urban	School-going	490	NR	NR	50.4%	NR	80.6%
Santina 2012 [59]	Lebanon	Urban	School-going	389	NR	74.3%	22.8%	34.6%	NR
Eryilmaz 2009 [80]^g^	Turkey	Unclear	School-going	1951	NR	72.2%	26.1% (of 1408)	NR	11.9% (of 1408)
Reis 2011 [27]	Turkey	Urban	Mix	310	NR	23.9%	NR	NR	NR

*PMS* Premenstrual symptoms/syndrome, *NR* Not reported

^a^Premenstrual symptoms or premenstrual syndrome

^b^Including dysmenorrhea

^c^Swelling or bloating

^d^Fatigue or dizziness

^e^Headaches and fatigue reported among girls with dysmenorrhea who agreed to provide more information

^f^Headaches and fatigue reported among girls with PMS

^g^Headaches and fatigue reported among girls with dysmenorrhea

### Social impacts

Activities of daily living or daily routines were restricted by menstruation among a quarter of girls in rural India [63], a third of female students in Brazil [58] and Egypt [44], and among 60.0% of slum dwellers in India [64]. In urban Sri Lanka, schoolgirls with premenstrual syndrome had significantly more disruptions to their daily routines than those without [43]. Daily activities are further limited by taboos related to what and who menstruating girls are able to come in contact with. Menstruating girls in India and Nepal are sometimes limited from entering kitchens or bedrooms to ensure menstrual blood does not contaminate food or others [1, 5, 11, 12, 63, 65]. Household work such as cooking was often cited as ‘not allowed’ for menstruating girls in India [1, 12, 63], Kenya [53], and Nepal [5]. Female students from a mix of rural and urban settings in India reported limitations on who they could touch while menstruating [12, 18]. Other social limitations frequently reported include avoiding physical or social activities (e.g., sports and functions), abstaining from religious activities or missing school (Table 6).

**Table 6:** Social impacts of menstruation

First author, Year	Country	Setting	School status	N	Avoid physical or social activities^a^ (%)	Abstain from religious activities (%)	Miss school or work (%)
East Africa							
Zegeye 2009 [21]^b^	Ethiopia	Mix	School-going	407	NR	NR	48.2%
Boosey 2014 [16]	Uganda	Rural	School-going	140	NR	NR	61.7% (of 133)
North Africa							
Abd El-Hameed 2011 [44]	Egypt	Mix	School-going	160	54	NR	NR
West Africa							
Adinma 2009 [26]	Nigeria	Urban	School-going	550	NR	NR	4.5%
Ajah 2015 [42]	Nigeria	Urban	School-going	482	NR	NR	12.2%
Aniebue 2009 [40]	Nigeria	Urban	School-going	495	37.6%	NR	2.0%
Oche 2012 [14]	Nigeria	Urban	School-going	122	4.9%	43.4%	NR
South America							
Pitangui 2013 [58]^b^	Brazil	Urban	School-going	127	NR	NR	30.7%
South Asia							
Dambhare 2012 [32]^b^	India	Mix	School-going	561	NR	NR	13.9%
Juyal 2013 [65]	India	Mix	School-going	453	8.6%	87.4%	NR
Thakre 2011 [12]	India	Mix	School-going	387	NR	44.7%	5.2%
Rana 2015 [63]	India	Rural	Mix	400	28.0%	53.2%	26.4%^c^
Bodat 2013 [67]	India	Rural	School-going	622	NR	NR	43.2%
Dasgupta 2008 [8]	India	Rural	School-going	136	36.3%	60.0%	13.8%
Kanotra 2013 [92]^b^	India	Rural	School-going	59	76.6%	NR	NR
Mudey 2010 [34]	India	Rural	School-going	300	NR	87.0%	NR
Shanbhag 2012 [10]	India	Rural	School-going	329	NR	94.2%	NR
Sudeshna 2012 [15]	India	Rural	School-going	190	NR	75.8%	37.9%
Chaudhuri 2012 [60]^b^	India	Unclear	School-going	128	53.5%	NR	25.8%
Sharma 2008 [64]	India	Urban	Mix	156	25.6%	NR	17.2% (of 116)^c^
Goel 2011 [9]	India	Urban	School-going	478	42.7%	76.2%	14.0%
Sharma 2003 [25]	Nepal	Urban	School-going	67	20.0%	NR	NR
Yasmin 2013 [13]	India	Urban	School-going	147	18.4%	90.5%	NR
Bobhate 2011 [7]	India	Urban	Unclear	241	24.1%	90.0%	NR
Ali 2010 [6]	Pakistan	Urban	Government school	425	67.3%	NR	NR
Ali 2010 [6]	Pakistan	Urban	Out-of-school	425	58.1%	NR	NR
Ali 2010 [6]	Pakistan	Urban	Private school	425	58.1%	NR	NR
Southeast Asia							
Lee 2006 [37]	Malaysia	Mix	School-going	2247	NR	NR	7.0%
Wong 2011 [47]^b^	Malaysia	Rural	School-going	984	58.6%	NR	18.1%
Wong 2010 [78]^b^	Malaysia	Urban	School-going	801	50.2%	NR	NR
Wong 2011 [48]	Malaysia	Urban	School-going	1076	61.5%	NR	NR
West Asia							
Poureslami 2002 [79]	Iran	Urban	School-going	250	33.0%	NR	15.2%
Santina 2012 [59]	Lebanon	Urban	School-going	389	NR	NR	41.4%

^a^Avoid or reduce physical activities (including playing and sports) or social activities (including functions and friendships with males)

^b^Among girls with dysmenorrhea

^c^Among school-going girls

Girls in Malaysia [38, 48] and Pakistan [6] reported restrictions on religious activities due to menstruation. Studies reporting the complete abstinence of religious activities come mostly from India; this practice was reported by 44.7–94.2% of girls interviewed [7–10, 12, 13, 15, 34, 60, 63, 64]. Education level had a significant influence on the practice of avoiding holy places in India (*p*-value < 0.05) [23]. Mothers interviewed in Nigeria revealed that they advised their daughters to refrain from praying during their periods [41]. This aligns with another study in Nigeria, in which 43.4% of girls reported abstaining from religious activities [14]. Health education interventions in Bangladesh [66] and India [19] did not result in significant declines in religious restrictions among girls during their periods.

When asked if girls can go to school while menstruating, 70.7% of girls in rural Nepal responded ‘no’ [5]. Actual absenteeism reported in various LMIC did not reach that level, instead ranging from 2.0% of urban-living girls in Nigeria [40] to 61.7% of rural-living girls in Uganda [16]. Focus groups in Malaysia revealed that dysmenorrhea may have a greater impact on school absenteeism for girls in urban settings than those in rural settings [38]. Dysmenorrhea was significantly associated with missing school among urban-living girls in Lebanon [59] as was pain severity among those in Brazil [58] (*p*-value < 0.05). Menstrual disorders in general was significantly associated with missing school among rural-living girls in India (*p*-value < 0.001) [67]. In Kenya, male teachers reportedly teased girls about menstruation when they returned to school after being absent for a few days [53]. Although the teachers denied this, they noted that they were concerned with girls being distracted in class [53]. Teachers interviewed in Ghana shared similar concerns about girls being distracted and missing school [68]. Girls, themselves, in India [60], Malaysia [47, 48], and Uganda [16] associated menstruation with poor academic performance and low grades.

A puberty education intervention with provision of sanitary pads in a non-randomized trial in Ghana significantly improved girls’ school attendance (*p*-value < 0.001) [69]. Alternatively, a randomized trial in Nepal [70] demonstrated that providing menstrual cups may improve convenience and mobility, and one in Kenya [71] demonstrated that they can reduce distractions associated with leakage and improve school attendance.

### How do girls respond to negative effects and what practices do they develop?

To address the physical impacts of menstruation described in Table 5, some girls reported using traditional medicine or remedies (Bangladesh [72], Brazil [58], India [63, 64, 73, 74], Malaysia [37, 47, 48, 75], Sri Lanka [43],) and others reported taking medication to relieve pain, often by self-medicating or consulting pharmacies (Bangladesh [72], Brazil [58], Egypt [44], India [60, 63, 64, 74], Iran [76], Malaysia [39, 47, 75], Nigeria [42], and Turkey [27, 77]). Consultation of health professionals for menstrual-related problems was minimal, generally reported by less than a fifth of girls (Bangladesh [72], Brazil [58], Ethiopia [21], India [60, 63, 64, 73, 74], Iran [76], and Malaysia [37, 47, 48],). However, one study did report that 69.8% of Indian girls with problems sought attention from a health professional [7]. Another study in India reported that 19.2% of girls with a problem never discussed it with anyone—a health professional, relative, or friend [64]. Girls in Bangladesh were significantly more likely to consult someone regarding their problems after participating in 12 health education sessions over the course of six months than at baseline (*p*-value < 0.01) [66].

General lack of adult guidance related to menstruation may contribute to the variation in basic hygiene management practices such as use of sanitary absorbents and bathing daily across LMIC (Table 7). Use of sanitary pads to absorb blood ranged from 2.0% of schoolgirls in rural Nepal [5] to 69.1–93.8% of urban-living girls in Nigeria [14, 20, 40, 41]. All but one study of girls in rural parts of seven Indian states reported greater proportions of girls using cloth compared to sanitary pads [1, 8, 10, 12, 15, 18, 29, 34, 63, 67, 78]. Sanitary pad use was significantly higher among urban-living girls in India [22] and Ethiopia [21] as was use of sanitary pads or new cloth among school-going girls in India [1] (*p*-value < 0.01). A quasi-experimental study testing village-based meetings for girls in India as a platform for disseminating health messages contributed to significant increases in the use of sanitary pads and a decrease in the reuse of cloth (*p*-value < 0.05) [3]. While fewer girls using sanitary pads in one study in rural India reported poor fit and rashes than did girls using cloth [63], the cost of sanitary pads were a concern for some girls questioned in other studies in India [12, 15, 34], Tanzania [61], and Uganda [16]. Almost all of the 102 urban-living girls questioned in Kenya preferred sanitary pads for their convenience and reliability, but nearly half used cloth or a combination of cloth and sanitary pads to save money [50]. Tissue paper and cotton were also cited as absorbents for girls in various LMIC, with tissue paper reported by as many as 37.1% of rural-living schoolgirls in Uganda [16].

**Table 7:** Menstrual hygiene management practices (%)

First author, Year	Country	Setting	School status	N	Use sanitary pads (%)	Use sanitary pads and cloth (%)	Use old or new cloth (%)	Use other material^a^ (%)	Bathe daily (%)
East Africa									
Zegeye 2009 [21]	Ethiopia	Mix	School-going	565	37.6%	NR	62.5%	NR	NR
North Africa									
Abd El-Hameed 2011 [44]	Egypt	Mix	School-going	160	NR	NR	NR	NR	100.0%^b^
El-Gilany 2005 [30]	Egypt	Mix	School-going	642	66.8%	NR	27.9%	5.3%	70.9%^b^
West Africa									
Iliyasu 2012 [41]	Nigeria	Urban	Mix	184	81.0%	NR	NR	NR	NR
Aniebue 2009 [40]	Nigeria	Urban	School-going	495	69.1%	NR	9.1%	21.8%	NR
Lawan 2010 [20]	Nigeria	Urban	School-going	371	93.8%	NR	6.2%	NR	NR
Oche 2012 [14]	Nigeria	Urban	School-going	122	86.9%	NR	9.0%	4.1%	NR
Boosey 2014 [16]^c^	Uganda	Rural	School-going	140	47.1%	NR	87.1%	37.1%	NR
South Asia									
Khanna 2005 [1]	India	Mix	Out-of-school	304	2.0%	NR	90.9%	0.3%	NR
Juyal 2013 [65]	India	Mix	School-going	453	38.4%	26.7%	34.9%	NR	63.6%
Khanna 2005 [1]	India	Mix	School-going	307	6.2%	NR	68.4%	0.7%	NR
Khanna 2005 [1]	India	Rural	Mix	281	3.2%	NR	92.2%	0.7%	NR
Rana 2015 [63]	India	Rural	Mix	400	39.0%	NR	61.0%	NR	NR
Bodat 2013 [67]	India	Rural	School-going	622	48.1%	NR	51.9%	NR	NR
Dasgupta 2008 [8]	India	Rural	School-going	160	11.3%	40%	48.8%	NR	NR
Kanotra 2013 [92]	India	Rural	School-going	323	89.5%	NR	10.5%	NR	NR
Mudey 2010 [34]	India	Rural	School-going	300	15.7%	NR	46.7%	NR	NR
Narayan 2001 [18]	India	Rural	School-going	327	1.7%	48%	82.5%	NR	NR
Shanbhag 2012 [10]	India	Rural	School-going	329	44.1%	21.2%	34.7%	NR	88.8%
Sudeshna 2012 [15]	India	Rural	School-going	190	13.2%	24.2%	62.6%	NR	NR
Thakre 2011 [12]	India	Rural	School-going	146	30.8%	NR	69.2%	NR	NR
Wasnik 2015 [72]	India	Rural	School-going	435	33.6%	9.2%	57.2%	NR	NR
Dhingra 2009 [29]	India	Rural	Unclear	200	NR	NR	NR	NR	0.0%^2^
Khanna 2005 [1]	India	Urban	Mix	330	4.8%	NR	69.0%	0.3%	NR
Goel 2011 [9]	India	Urban	School-going	478	NR	NR	NR	NR	92.9%
Nair 2012 [73]	India	Urban	School-going	3443	45.5%	38.2%	16.3%	NR	97.6%
Narayan 2001 [18]	India	Urban	School-going	292	8.3%	17.1%	72.2%	NR	NR
Omidvar 2010 [46]	India	Urban	School-going	350	68.9%	NR	19.1%	11.1%	81.7%^b^
Thakre 2011 [12]	India	Urban	School-going	241	60.6%	NR	39.4%	NR	NR
Yasmin 2013 [13]	India	Urban	School-going	147	82.3%	1.4%	16.3%	NR	85.7%
Bobhate 2011 [7]	India	Urban	Unclear	241	43.2%	NR	41.5%	15.4%	NR
Adhikari 2007 [5]	Nepal	Rural	School-going	150	2.0%	NR	98.0%	NR	4.0%
Ali 2010 [6]^c^	Pakistan	Urban	Government school	425	17.9%	NR	87.5%	3.9%	44.2%^b^
Ali 2010 [6]^c^	Pakistan	Urban	Out-of-school	425	13.2%	NR	81.0%	6.6%	45.9%^b^
Ali 2010 [6]^c^	Pakistan	Urban	Private school	425	33.8%	NR	62.6%	4.4%	45.2%^b^
West Asia									
Poureslami 2002 [79]	Iran	Urban	School-going	250	NR	NR	NR	NR	66.0%^b^

*NR* Not reported

^a^Other materials include various types of tissue and cotton

^b^Proportion of girls that bathe at all during their period

^c^Multiple responses accepted for materials used

A minority of girls in Egypt [30] and India [12, 46], no more than one in five, compared to 56.5% of girls in Nigeria [20] changed absorbents while at school. Most girls in Egypt [30] and Uganda [16] felt schools lacked privacy and most in India [12] and Nigeria [20] preferred to change materials at home. Insufficient latrines, water supplies and disposal infrastructure further presented a barrier for students in India [15, 67], Tanzania [61], and Uganda [16] to manage their periods at school.

Methods for disposing of materials beyond throwing them away with other trash included burning, burying, and flushing materials. Very few (2.5%) girls in Egypt dispose of absorbents by burning them compared to 17.0–76.0% in India [12, 63, 73] and Nigeria [14, 40]. Indian girls in rural settings were significantly more likely to report burning materials than those in urban settings (*p*-value < 0.05) [22]. Of those who reuse cloth, drying washed materials in sunlight rather than in hiding varied from 30.7% of girls in urban Pakistan [6] to 44.3–72.4% of schoolgirls in India [10, 12, 15, 18, 73]. School-based health education in India led to significant improvements in washing cloth with soap, in drying them in the sun and in disposing of them safely [17].

Reported bathing practices in India ranged from all 200 rural-living girls in one state abstaining during menstruation [29] to nearly all 3,443 girls in urban areas of another state bathing daily [73]. One study found that the practice of daily bathing was significantly higher among urban-living girls than rural-living girls (*p* < 0.05) [65], and another found that both a regular source of water and a private bathroom exclusive to a family had significant relationships with taking a daily bath (*p* < 0.001) [13]. In Turkey [27] and Nigeria [20], 11.9% and 72.5% of urban-living girls reported increasing the number of baths they take. Girls in rural Kenya revealed that they wanted to bathe more frequently during their period, but were concerned about using limited water supplies and feared revealing to their family that they were menstruating [51]. A quasi-experimental study involving 698 girls in Iran showed that those who participated in 10 two-hour teacher-led sessions on pubertal changes engaged in regular bathing more than those who did not, and the difference was significant (*p*-value < 0.01) [79]. Another quasi-experimental study in Egypt found a significant increase in the number of girls bathing daily during their periods after participating in four 30–45 min health education sessions focused on menstruation (*p*-value < 0.001) [80]. Four studies in India defined satisfactory cleaning of genitalia to mean washing two or more times a day while menstruating; one-third to three-quarters of girls met this criterion [12, 13, 29, 34]. Unsatisfactory cleaning was significantly higher among rural-living girls than those in urban parts [22]. In a study evaluating the impact of school-based health education, the proportion of girls using soap to clean their genitalia significantly increased from 30.0 to 94.3% (*p*-value < 0.01) [17].

Girls in Mexico who had previous knowledge of the physiology of menstruation were significantly more likely to know what was happening in their bodies and what to do, in terms of hygiene management, upon reaching menarche (*p*-value < 0.001) [24]. For schoolgirls in Jordan, being prepared prior to menarche resulted in more positive attitudes, and attitude had a significant positive correlation with practices (*p*-value < 0.05) [36]. Schooling status among girls in Iran also had a significant positive correlation with practices (*p*-value < 0.01) [6, 66].

## Conclusion

An important limitation of this review is that vague measures are often used to describe the menstrual experiences of girls, which impede data aggregation and direct comparisons. For instance, studies used different yardsticks for adequate or inadequate knowledge, and used the terms premenstrual syndrome and dysmenorrhea loosely. Further, many studies had small sample sizes and relied heavily on self-report. Some studies had low response rates due to discomfort or limitations to discussing menstruation. Another limitation is that a majority of relevant data from included studies come from a limited number of countries and are not representative of all LMIC. Among the included countries and across all LMIC is great cultural variation, and the results presented here should be considered in light of these unique perspectives. Despite these limitations, the evidence presented allows for the following conclusions:

- Substantial numbers of girls in many countries have knowledge gaps and misconceptions about menstruation. This leaves them unprepared when they reach menarche and causes fear and anxiety.
- Mothers, other female relatives and female peers are their main sources of information and advice on menstruation. The information they receive, however, is not always timely nor adequate. Only some have access to additional information from sources such as mass media and the Internet.
- Girls experience a variety of symptoms during menstruation—pain, headaches and fatigue. These symptoms combined with taboos result in their not being able to participate in household, school, or social activities.
- Very few girls seek health care when they experience menstrual health problems. If anything, they may resort to household remedies.
- Girls in poor urban and rural communities of LMIC are less likely to obtain and use sanitary pads. Instead, they use materials made at home with scraps of old cloth, cotton, paper, etc. Lack of privacy, access to clean water and functional toilets make it harder for them to manage their periods.

It is clear that far too many girls across LMIC are struggling with nearly complete ignorance of their normal biological maturation and its consequences, and when they do receive education, still struggle with inadequate sanitary materials and insufficient physical and emotional support. Although there is no convincing evidence that poor menstrual hygiene management leads to ascending reproductive tract infections [81] or causes lasting sequelae, this review underscores that coming to terms with menarche and navigating the shame and practical challenges associated with its management may cause girls great anxiety and sadness. There remains a need for further research into the physical, mental and social impacts of such distress. For example, being unprepared for menarche, being excluded and shamed during monthly periods, being hindered in self-care and uncared for when unwell, undermines a girl’s sense of being in charge of her life, her sense of self-worth and her sense that the individuals and institutions around her are responsive to her needs. The huge and lasting impacts this can have on girls’ lives remain to be studied.

In the short term, however, there are intervention studies that demonstrate the ability to improve girls’ menstrual knowledge and hygiene management. Health education interventions like school-based sessions tested in India have resulted in improved understanding post-intervention [17], and similar programs in Egypt [80] and Iran [79] have improved the bathing practices of girls during their periods. Additionally, a quasi-experimental study in India that involved training of medical officers and providing reference tools led to statistically significant improvements in their case management of menstrual health problems for female patients between the ages of 15 and 24 [82].

Some exciting initiatives led by academics, international agencies and the private sector are also under way. Educating and encouraging parents to communicate with their daughters and sons about puberty and menstruation is being implemented by the Families Matter Program [83]. A five-year initiative by Columbia University has launched locally designed puberty booklets for girls and for boys in Tanzania, Ghana, Ethiopia, and Cambodia; these have been embraced by the Ministries of Education and of Health in all four countries [84]. Save the Children has also developed workbooks, modeled on those by Columbia University, for girls and boys in Nepal, Uganda and Malawi and is carrying out puberty education programs in multiple countries [85]. Similar efforts, such as CycleSmart^TM^ [86] and GrowUp Smart [87], are being implemented by Georgetown University’s Institute of Reproductive Health in Rwanda and Guatemala. In 2014, UNESCO published a policy booklet with guidance to improve school administrators and teachers’ abilities to educate and support girls and boys in classrooms [88]. Procter & Gamble, a major producer of sanitary products, has launched communication programmes in several of the countries where it sells products with marketing approaches aimed at legitimizing family discussion of menstruation, and engaging and educating girls while building their self-esteem [89]. During its Celebrating Womanhood event in 2013, the Water Supply & Sanitation Collaborative Council defined menstrual hygiene as a priority and outlined a 3-pronged approach that includes breaking the silence around this topic, ensuring hygienic management, and identifying mechanisms for safe reuse and disposal of materials [90]. Linking menstruation education with efforts to improve water, sanitation and disposal facilities in schools has also been actively promoted and implemented by UNICEF at the country and global levels [91].

While these initiatives are important and promising first steps, greater uptake and commitment is needed to fulfill the rights of girls related to menstrual knowledge, health and hygiene. Concerted multi-level efforts are required to achieve this. At the individual level, girls and boys need to be educated about puberty. At the family level, girls need support during their menstrual cycles. At the community level, we must improve access to sanitary products, running water, functional toilets and privacy. We need competent and caring health care workers who can respond to girls’ questions and concerns, and to provide care when they have menstrual health problems. Finally, we need leaders who can change the perception of menarche and menstruation to one of normalcy and promise rather than of shame.

## Abbreviations

LMIC: Low- and middle-income countries
MHM: Menstrual hygiene management

## Footnotes

1 Only descriptive studies with quantitative results are included in Tables 2, 3, 4, 5, 6 and 7.
